# rTMS For PTSD: Induced Merciful Oblivion or Elimination of Abnormal Hypermnesia?

**DOI:** 10.1155/2006/793256

**Published:** 2006-11-27

**Authors:** Simone Rossi, Stefano F. Cappa, Monica Ulivelli, Alberto De Capua, Sabina Bartalini, Paolo M. Rossini

**Affiliations:** ^1^Dipartimento di NeuroscienzeSezione NeurologiaUniversità di SienaPoliclinico le ScotteViale BracciI-53100SienaItaly; ^2^Centro di Neuroscienze CognitiveUniversità Salute-Vita S. RaffaeleDIBIT Via Olgettina 58I-20132MilanoItaly; ^3^Dipartimento di NeuroscienzeSezione PsichiatriaUniversità di SienaPoliclinico le ScotteViale BracciI-53100SienaItaly; ^4^AFaR.- Dipartimento di NeuroscienzeS.Giovanni Calibita FatebenefratelliIsola TiberinaI-00186RomaItaly; ^5^Clinica NeurologicaUniversità Campus BiomedicoVia Longoni 71RomaItaly

**Keywords:** PTSD, TMS, rTMS, amnesia, episodic memory

## Abstract

Neuroimaging studies and experimental data suggest that symptoms of posttraumatic stress disorder (PTSD) are associated with dysfunctions of neural circuits linking prefrontal cortex and the limbic system that have a role in autobiographic episodic memory. High-frequency repetitive transcranial magnetic stimulation (rTMS) of the right dorsolateral prefrontal cortex (DLPFC) has been suggested to be beneficial to patients with PTSD, transiently alleviating re-experiencing as well as avoidance reactions and associated anxiety symptoms. In healthy humans, converging evidence suggests that rTMS of the right DLPFC interferes with episodic memory retrieval. Hence, we hypothesize that daily applications of rTMS in PTSD patients may reduce access to the set of autobiographical stored events, that, if re-experienced, may cause the overt PTSD symptoms.

## 1. The concept of autobiographic episodic memory

Seminal, personally experienced, events occurring along our life-span are stored somewhere in the brain. They can be intentionally retrieved, or can emerge unexpectedly, in each moment of everyday life. Normally, these stored events can give rise to joy or sadness, or even anxiety: this is what is referred as “autonoetic consciousness” [13,37]. Such autobiographic traces are the building blocks of episodic memory, a concept indicating a complex set of human cognitive processes that allows the encoding, storage, and intentional recollection (retrieval) of unique events associated with the context in which they occurred [1]. Therefore, the correct functioning of episodic memory mechanisms is pivotal to form the conscious story of our existence [38]. Traditional neuropsychology has looked at amnesia as the most typical dysfunction of the episodic memory system [36].

## 2. Post-traumatic stress disorder (PTSD) as a dysfunction of autobiographic memory

The intrusive, sometimes iterative, recollection of personally experienced unpleasant past events may cause, in some people, a disabling clinical syndrome known as posttraumatic stress disorder (PTSD), characterized by emotional numbing and withdrawal, psychological and physiological hyperarousal, as stated in

ISSN 0953-4180/06/$17.00 © 2006 – IOS Press and the authors. All rights reserved the DSM-IV. Depression, anxiety and avoidance reactions may accompany the re-experiencing of such autobiographical target events. Functional neuroimaging and neurophysiological techniques have identified significant neurobiologic changes in the brain of people suffering from PTSD, and these changes take place in areas known to be important in both stress and memory (see [24]). Such advances in cognitive neuroscience have begun to unveil intriguing relationships between memory disturbances and affective disorders (see [17]) both in vivo and at experimental levels. By measuring brain regional cerebral blood flow or metabolism, it has been suggested that dysfunctions of neural circuits linking prefrontal cortex, anterior-cingulate, amygdala and limbic system are detectable in PTSD patients [7,35], together with hyperexcitability at cortical levels, probably due to dysfunction of cortico-cortical inhibitory networks [6]. These functional abnormalities are even more apparent using symptom provocation paradigms, during which dysfunctions of prefrontal cortex and limbic system are associated with the retrieval of the target event or with the overt PTSD syndrome [2,19,35].

Experimentally derived data indicates that acute stress affects brain activity by promoting neuroplastic long-term changes of synaptic efficacy sustained by long-term potentiation (LTP) of excitatory synapses. LTP, which refers to the enduring facilitation of the communication between two neurons following the activation of the synapses by which they are interconnected, is believed to be the substrate for both explicit and implicit learning and memory processes (see [14, 22]). Environmental, physical, and mental stressful events are factors able to promote LTP changes [15] taking place in brain structures and systems that are relevant for both memory processing and the stress response [3]. Hence, it can be postulated that such neuroplastic changes could represent the synaptic correlates of the maintenance of traumatic autobiographic memories that cause PTSD [6].

## 3. PTSD can be alleviated by repetitive transcranial magnetic stimulation (rTMS)

In a recent double-blind, placebo-controlled study that appeared in the American Journal of Psychiatry, Cohen and coworkers [8] applied, in a cohort of PTSD patients, trains of high-frequency (10 Hz) rTMS for 10 days over two weeks. TMS is currently the only technique that can non-invasively produce a transient dysfunction of the targeted cortical area [25,41]. In the Cohen et al. study, the targeted brain region was the right dorsolateral prefrontal cortex (DLPFC). They found a significant, relatively long-lasting, behavioral improvement regarding both re-experiencing and avoidance, together with alleviation of anxiety symptoms.

The results of the Cohen et al. study [8], which probably opens new neuromodulatory treatment avenues for PTSD, have been interpreted according to the hypothesis of rTMS-induced long-lasting activation of the hypofunctioning right DLPFC. The authors hypothesized that this "activation" would be associated with improvements of the hypofunctionig hypothalamic-pituitary-adrenal axis, with enhanced activity of the depressor area of autonomic responsiveness, or with an increased functional suppression of the amygdala. A further speculation was that high-frequency rTMS of the right DLPFC might have improved functional connectivity mechanisms in PTSD patients. These interesting conjectures are certainly worthy of consideration: Future studies should be aimed to evaluate in patients with PTSD treated by daily rTMS changes in hormonal blood levels, autonomic function, and functional coupling of regional electroencephalographic cortical rhythmicity.

## 4. rTMS and episodic memory

Another hypothesis, not mutually exclusive with that from the Cohen et al. study, merges recent evidence from episodic memory studies with PTSD pathophysiology to account for rTMS-induced beneficial effects. To better explain this novel hypothesis in the following paragraph, recent data on TMS and episodic memory should be briefly discussed.

First, we should consider that, although mechanisms of action of high-frequency rTMS are not yet fully understood, it is quite clear that transient interference of brain function is more likely to occur than behavioral improvements [27,41]. Of note, the term "virtual lesion" has been coined to depict rTMS effects on behavior [25]. Recent data also suggest that high-frequency rTMS may promote neuroplastic LTP-mediated changes at cortical levels [10].

On the basis of this "interferential" approach, high-frequency rTMS (20 Hz) applied on the right DLPFC (i.e., the same target region of the PTSD study) in healthy humans has been used to prove the existence of a causal relationship between this brain region and retrieval processes during episodic memory tasks: Under rTMS, but not during sham stimulation, subjects made significant recognition errors of previously encoded visual scenes [28,29] or verbal items [12,33], thus demonstrating that the correct functioning of episodic memory system actually relies on PFC function, with a clear-cut right functional prevalence for retrieval mechanisms of complex visuospatial pictures representing real-life environments [28,29]. These findings are in line and extend previous correlational results of many neuroimaging studies based on regional changes of cerebral blood flow and metabolism [4,5,11]. While these results have been collected in experimental studies dealing with recently memorized information, there is now ample evidence that the prefrontal cortex is engaged also by the retrieval of remote autobiographical memories [26], the types of memories that are probably dysfunctional in PTSD.

## 5. An alternative hypothesis to explain rTMS effects in PTSD

For a single and brief train of stimulation, as would be applied in memory studies [12,28,29,33], the interferential effect is obviously short-lived, lasting a matter of seconds (i.e., sufficiently long for the subject to answer after the elaboration of the presented visual stimulus). In patients with PTSD, daily applications of high-frequency rTMS to the right DLPFC (which, as previously described, is functionally-necessary for autobiographical retrieval mechanisms) might have induced, probably through temporal summation, a prolonged dysfunction of the brain mechanisms underlying the episodic retrieval for personally experienced real-life events (i.e., the autonoetic consciousness).

Interestingly, patients with PTSD are very suitable candidates for rTMS interference during long-term memory tasks, due to their functional hypoactivation of the right DLPFC [2,19] and to the relatively frequent association of other memory dysfunctions with this psychiatric syndrome [16,40]. Assuming that LTP in brain circuitries relevant for recall represents the synaptic correlates of the maintenance of traumatic autobiographic memories that cause PTSD [6], it can be hypothesized that rTMS of the DLPFC interferes with such mechanisms. Therefore, daily interference with rTMS may produce a sort of amnesia for the distressing life event that is the prime mover for PTSD.

Looking at PTSD symptoms from a different perspective, but still in the frame of a dysfunction of neural circuitry underlying memory processes, the "abnormal" autobiographic retrieval might be even regarded as sharing behavioral similarities with the automatic hypermnesia as the one seen in schizophrenic or in autistic savant patients [20,23]. Along this vein, an intriguing hypothesis is that the abnormal and intrusive reiteration of the target autobiographical event might induce – while simultaneously being sustained by- synaptic changes linked with LTP mechanisms. In the long-term, these mechanisms may contribute, together with the partial loss of right DLPFC modulatory activity which is hypofunctioning in PTSD patients, to re-shape paradoxical fronto-temporal-limbic functional connections sustaining PTSD symptoms. Similar mechanisms of aberrant synaptic plasticity are known to take place in other neurological conditions such as stroke or chronic pain [30]. Notably, rTMS may have some transient beneficial effect both in stroke recovery and chronic pain treatment [18,21]. Through such a mechanism, daily rTMS may interrupt the intrusive reiteration of the target autobiographical event through a resetting of the DLPFC function.

In other words, whatever the mechanism, daily rTMS may temper to a more physiological level the intrusive, LTP-mediated access to the set of stored events, that, if re-experienced, cause the overt PTSD picture, thereby restoring a better-functioning autonoetic consciousness mechanism. If this memory-oriented hypothesis put forward to explain beneficial effects of rTMS in PTSD is true, then the classical manifestation of an episodic memory dysfunction (i.e., amnesia) could be regarded either as a merciful oblivion or as a sort of down-regulation of abnormal hypermnesia processes, resulting in symptom alleviation for these patients.

## 6. Questions for future research

How might these hypotheses be reliably addressed in future TMS research? The best approach is probably to start from experimental data. Fear conditioning is a form of associative learning between certain neutral stimuli (conditioned stimuli) and aversive events (unconditioned stimuli), and is considered to be a suitable animal model of PTSD [39]. A bulk of experimental studies based on the fear conditioning paradigm [9,31,32,34] converge to suggest that the acquisition and retrieval of an intense fear, mimicking a traumatic event and its repeated recollection, have an impact on LTP induction in the synapses of the dorsal hippocampus. Such induction may underpin the mechanism of memory deficits by stress or trauma re-experiencing in PTSD patients. Verifying how and how long these synaptic changes are modulated by rTMS (and if these changes are paralleled by behavioural improvement toward fear conditioning and response) seems an appropriate "first step" to better understand the effects of rTMS on human PTSD.
